# Association between the perceived value of adopting new behaviors and depressive symptoms among older adults

**DOI:** 10.1038/s41598-024-55301-4

**Published:** 2024-02-25

**Authors:** Chiharu Nishijima, Osamu Katayama, Sangyoon Lee, Keitaro Makino, Kenji Harada, Masanori Morikawa, Kouki Tomida, Ryo Yamaguchi, Kazuya Fujii, Yuka Misu, Hiroyuki Shimada

**Affiliations:** 1 Center for Gerontology and Social Science, National Center for Geriatrics and Gerontology, Obu, Aichi 474-8511 Japan; 2 Japan Society for the Promotion of Science, Chiyoda-ku, Tokyo, 102-0083 Japan; 3 Obu Center for Dementia Care Research and Practices, Obu, Aichi 474-0037 Japan

**Keywords:** Behavior, Community-dwelling older adults, Depressive symptom, Perceived value, Preventive measure, Health care, Risk factors

## Abstract

Early preventive measures against depression have become important with unprecedented global aging. Increase in one’s perceived value (PV) may correspond to better mental health outcomes. This cross-sectional observation study aimed to clarify whether the PV of adopting new behaviors is associated with depressive symptoms. The participants were 5266 community-dwelling older adults aged ≥ 65 years. We developed a questionnaire to measure the PV of adopting new behaviors, specifically activities beneficial for preventing depressive symptoms (physical, cognitive, and social activities) in older adults. The questionnaire asked whether adopting the ten selected behaviors was valuable. The scores were added, and the total score ranged from − 20 to 20. The odds ratios (OR) of depressive symptoms were calculated using binomial logistic regression according to the PV score quartiles. Depressive symptoms were reported by 595 (11.3%) participants. After adjusting for potential confounders, higher quartiles of PV scores were significantly associated with lower prevalence of depressive symptoms: vs Q1; Q2 OR 0.76 (95% confidence interval: 0.59–0.97); Q3 0.67 (0.51–0.87); Q4 0.54 (0.40–0.73) (*P* for trend < .001). Having a higher PV of adopting new behaviors may prevent depressive symptoms among older adults. Healthcare professionals need to pay attention to poor value orientation among older adults.

## Introduction

Depression is a common mental health disorder among the older population and a major cause of adverse life and health outcomes, such as low quality of life, worsening of comorbid illnesses, functional and cognitive decline, and the development of disabilities^1–4^. A pooled analysis of 24 studies involving community-based older adults reported that the prevalence of depressive disorders among older adults is 17.1%^5^. With unprecedented global aging, early preventive measures are particularly important.

Engaging in regular physical, cognitive, and social activities is beneficial for preventing frailty and depressive symptoms^6–14^. Systematic reviews and meta-analyses of longitudinal observations and intervention studies have shown a protective effect of physical activity against depressive symptoms^9,10^. Physical activity can contribute to the natural production of endorphins (endogenous opioid neuropeptides including serotonin, dopamine, and noradrenaline), which help reduce depressive symptoms^15^. The relationship between cognitive activity and reduced depressive symptoms has been reported in some observational studies. However, other than the commonality that cognitive decline is accompanied by depressive symptoms, the underlying mechanisms remain unclear^8,9,11^. Various definitions of social aspects (e.g., social activity, interaction, and networks) have been found to have a protective effect on depression^8,12–14^. However, inducing behavior change is generally challenging^16^.

The role of values has gained attention in recent years^17–20^. Values are defined as cognitive beliefs about what is important, good, and worthwhile for individuals or societies, and what is not^17^. Personal values determine what we consider important and worthwhile in life and are widely believed to serve as behavioral and motivational guides^21,22^. Studies on values in relation to health have revealed that values correlate with subjective well-being, life satisfaction, quality of life, and mental health disorders, such as depression^17–20^. Research has assessed values using Schwartz's theory of basic human values and demonstrated that openness to change values are negatively correlated with depressive symptoms in adolescents^18,20^. Openness to change reflects one’s readiness for change; thus, how people perceive to be valuable for change may be associated with depressive symptoms. In the case of health behaviors such as physical, cognitive, and social activities, perceived value (PV) may be associated with engagement in those activities; in turn, an increase in the PV of adopting new behaviors could correspond to better long-term mental health outcomes.

Intervention trials have shown that perceptions can be modified through experience or appropriate education^23,24^. Cognitive behavioral therapy, a well-known psychological treatment for psychiatric disorders, involves changing patients’ perceptions and thinking patterns through psychoeducation, homework, behavioral activation, and problem-solving, used alone or in combination^25^. This indicates that identifying those with a low PV of adopting new behaviors and enhancing their PV through education or other means may contribute to behavioral change and promoting mental health, consequently preventing depressive symptoms.

To date, the relationship between the PV of adopting new behaviors and depressive symptoms remains unclear. Therefore, this study aimed to clarify whether the PV of adopting new behaviors is associated with depressive symptoms in community-dwelling older adults.

## Methods

### Study design and participants

This cross-sectional study enrolled community-dwelling older adults from a sub-cohort of the National Center for Geriatrics and Gerontology Study of Geriatric Syndromes, which is a population-based national cohort study that aims to establish a screening system for geriatric syndromes and validate evidence-based interventions to prevent them^26^. This sub-cohort included older adults aged ≥ 65 years in March, 2017, who resided in Tokai city, a residential suburb in Nagoya, Japan. Invitation letters for baseline health checkup were sent to 20,248 independent residents aged ≥ 65 years who were not hospitalized, in residential care, or participating in another study. Written informed consent was obtained from all participants before they participated in the study. The study protocol was conducted in accordance with the Declaration of Helsinki and approved by the ethics committee of the National Center for Geriatrics and Gerontology (No. 1440-5).

A total of 5,563 older adults in Tokai City participated in health checkups. For our analysis, we excluded participants with incomplete responses to questions about their perceptions toward adopting new behaviors (n = 98), those with a history of dementia (n = 11), Parkinson’s disease (n = 17), depression (n = 97), severe cognitive decline [Mini-Mental State Examination scores (MMSE) < 18 (n = 21)]^8,27^, those with a lack of independence in basic activities of daily living [such as eating, bathing, grooming, walking, and stair-climbing (n = 13)], and those with functional disabilities as certified by the national long-term care insurance system (n = 27). We also excluded patients with incomplete responses to depressive symptoms (n = 13). Finally, 5266 participants were included in the final analysis. All assessments were performed by well-trained study assistants at community centers.

### Measurements

#### Depressive symptoms

Depressive symptoms were assessed using the 15-item Geriatric Depression Scale (short form)^28^, which has demonstrated adequate validity as a measure of depression among older adults. This short version was developed by selecting the 15 items that had the highest correlation with depressive symptoms and was validated with a high overall correlation with the original version (*r* = 0.89)^29^, A cut-off score of ≥ 6 was confirmed to have a sensitivity of 82% and specificity of 75% compared with a structured interview for clinical depression^30^.

#### PV questionnaire

There is no questionnaire to measure the PV of adopting new behaviors. The questions were developed by consulting gerontology experts who had a Ph.D. in health and social science or rehabilitation science to assess whether participants perceived to be valuable for adopting new health behaviors. Ten behaviors were selected, focusing on physical, cognitive, and social activities that relate to reduced geriatric syndrome including depressive symptoms. These activities are often closely interrelated; for example, physical activities include exercising or sports, which can be performed alone or with other people, and team sports, which provide social interaction. Some cognitive activities, such as participating in group discussions, also have social aspects. Thus, behaviors were selected to include physical and cognitive activities as well as social interactions that accompany reported health behaviors such as playing ground golf, taking enrichment classes, and attending a community meeting^8,31^. In addition, because the sense of economy, a lack of occupation, and loneliness, may influence one’s participation in activities, the ten behaviors included starting a paid activity, having a job, and some behaviors related to social networking and contacts that are opposite of loneliness^6,32,33^. We asked participants whether adopting the following behaviors was valuable: (1) making a new friend, (2) making a new chatting acquaintance, (3) starting a new paid activity, (4) belonging to a new group, (5) interacting with people of different ages and sexes, (6) starting a new exercise or sport, (7) starting a new cognitive activity, (8) starting to participate in social activities, (9) increasing opportunities to talk to new people, and (10) starting a new job with no experience. For each question, participants chose their responses from “not at all = − 2,” “not so much = − 1,” “somewhat valuable = 1,” and “very valuable = 2.” These values were added to obtain the total score that ranged from − 20 to 20.

### Potential confounding factors

The potential confounders of depressive symptoms were selected based on previous studies^30,34–38^. The demographic characteristics included age, sex, years of education, and living status (whether one lived alone). We also included annual household income, alcohol consumption, and smoking as demographic characteristics because they may be associated with depressive symptoms^39,40^. Health factors included history of heart disease, stroke, hypertension, hyperlipidemia, diabetes, body mass index, cognitive status (MMSE), and slow gait. Pain was also included as a health factor because an association between pain and depression has been reported^41^. Data on these factors were obtained through interviews and measurements conducted by nurses and trained staff. Walking speed was measured by asking the participants to walk for 6.4 m at a comfortable speed on a flat, carpeted surface with no shoes, and a walking speed of 2.4 m in the middle of the whole walkway was measured. A digital stopwatch was used to automatically determine when participants walked past infrared sensors at the start and end of the measurement area. A walking speed less than 1.0 m/s was defined as a slow gait^37^. Physical, cognitive, and social activities were included as an engagement in activities. Engagement in physical and cognitive activities was assessed using a leisure activity score that consisted of 11 physical activities (playing tennis or golf, swimming, bicycling, dancing, participating in group exercises, playing team games (e.g., bowling), walking for exercise, climbing more than two flights of stairs, doing housework, and babysitting) and 6 cognitive activities (reading books or newspapers, writing for pleasure, doing crossword puzzles, playing board games or cards, participating in organized group discussions, and playing musical instruments)^42^. Participants reported their frequency of engagement as “daily,” “several days a week,” “once weekly,” monthly,” “occasionally,” or “never,” and the scores were calculated according to frequency, ranging from 0 to 77 for physical activity and 0 to 42 for cognitive activity. Social activities were assessed based on how many of the following behaviors the participants engaged in: (1) going out for tea or dining with friends, (2) shopping with friends, (3) having conversations with anyone, and (4) having paid work^8,13^.

### Statistical analysis

Participants’ characteristics were compared between participants with and without depressive symptoms using unpaired t-tests or Pearson’s chi-square tests. Internal consistency was evaluated using Cronbach’s alpha coefficient and was considered adequate (> 0.70)^43^. The test–retest reliability of each component was assessed using the intraclass correlation coefficient (ICC). Based on the 95% confidence intervals (CIs) of the ICC estimate, values less than 0.5, between 0.5 and 0.75, between 0.75 and 0.9, and greater than 0.90 are indicative of poor, moderate, good, and excellent reliability, respectively^44^. The participants were divided into quartiles of PV scores. The variables were compared using one-way analysis of variance and Pearson’s chi-square test. The association between the score and the presence of depressive symptoms was examined using binomial logistic regression analysis with crude and adjusted models for potential confounding factors. Data were presented as odds ratios (ORs) with 95% CIs. All analyses were performed using IBM SPSS Statistics, version 28 (IBM Japan, Tokyo). Statistical significance was set at a two-tailed probability of *P* < 0.05.

## Results

Participants enrolled in this study were 5,266 older adults (mean age with standard deviation (SD) = 74.1 ± 5.6 years; women = 55%). The differences in characteristics between participants with and without depressive symptoms are shown in Table 1. Of the 5266 participants, those who had depressive symptoms (595; 11.3%) were older, had fewer years of education and lower annual household income, lived alone, consumed less alcohol, smoked more, had more heart diseases, stroke, hyperlipidemia, pain, and cognitive decline, and had lower body mass index, slower gait, and less engagement in social activities (P < 0.05).

**Table 1:** Characteristics of participants with or without depressive symptoms.

	Without depressive symptoms n = 4671 (88.7%)	With depressive symptoms n = 595 (11.3%)	*P* value
Demographic characteristics			
Age, years	73.9 ± 5.5	75.5 ± 6.1	< 0.001*
Sex, female (%)	2586 (55.4)	308 (51.8)	0.097^†^
Education, years	11.7 ± 2.3	11.2 ± 2.3	0.008*
Annual household income, ≥ 3 million yen (%)	2214 (47.4)^‡^	192 (32.3)^§^	< 0.001^†^
Living alone, yes (%)	605 (13.0)^§^	123 (20.7)^‡^	< 0.001^†^
Alcohol consumption, yes (%)	1966 (42.1)^‡^	224 (37.6)^§^	0.038^†^
Smoking, yes (%)	282 (6.0)^§^	60 (10.1)^‡^	< 0.001^†^
Health factors			
Heart disease, yes (%)	853 (18.3)^§^	143 (24.0)^‡^	< 0.001^†^
Stroke, yes (%)	254 (5.4)^§^	64 (10.8)^‡^	< 0.001^†^
Hypertension, yes (%)	2242 (48.0)^§^	311 (52.3)^‡^	0.050^†^
Hyperlipidemia, yes (%)	1797 (38.5)^§^	255 (42.9)^‡^	0.039^†^
Diabetes, yes (%)	626 (13.4)	97 (16.3)	0.052^†^
Pain, yes (%)	1955 (41.9)^§^	329 (55.3)^‡^	< 0.001^†^
BMI, kg/m^2^	23.3 ± 3.1	23.0 ± 3.3	0.029*
Cognitive decline, MMSE < 24 (%)	311 (6.7)^§^	89 (15.0)^‡^	< 0.001^†^
Slow gait, < 1.0 m/s (%)	986 (21.1)^§^	220 (37.0)^‡^	< 0.001^†^
Engagement in activities			
Physical activity, LA score	16.0 ± 8.3	13.2 ± 7.5	0.054*
Cognitive activity, LA score	11.6 ± 6.1	9.5 ± 5.7	0.194*
Social activity, number	2.7 ± 0.9	2.1 ± 1.0	< 0.001*
Depressive status			
GDS score, point	1.9 ± 1.5	7.8 ± 1.9	< 0.001*

BMI, Body Mass Index; MMSE, Mini-Mental State Examination; LA score, leisure activity score; GDS, Geriatric Depression Scale-Short Form.

*P-values reported from unpaired t-test.

^†^*P*-values obtained by Pearson’s chi square test.

^‡^Statistically significant association with adjusted standardized residual > 1.96 [*P* < 0.05].

^§^Statistically significant association with adjusted standardized residual < 1.96 [*P* < 0.05].

The internal reliability of the PV questionnaire, assessed using Cronbach’s alpha, was 0.925. The inter-item correlations were high for the scale (item test), and the coefficients ranged from 0.64 to 0.78. The ICC (1, 2) for the total score was 0.79 (95% CI 0.51–0.92) and indicated moderate to excellent test–retest reliability. The mean ± SD scores for the PV items ranged from 0.19 ± 1.34 to 0.98 ± 1.13 (Table 2). The mean, median, and interquartile ranges of the total scores were 6.38, 8, and 0–13, respectively. Each question item was significantly negatively associated with the GDS score, with correlation coefficients ranging from − 0.146 to − 0.216 (All, *P* < 0.001). All of the question items significantly correlated with physical, cognitive, and social activities (All, *P* < 0.001).

**Table 2:** Mean and median scores of each PV questionnaire item and correlation between items and GDS score, physical, cognitive, and social activities.

	Mean ± SD	Median (25th–75th percentile)	Spearman’s correlation coefficient ^†^			
			GDS score	Physical activity	Cognitive activity	Social activity
Making a new friend	0.98 ± 1.13	1 (1–2)	− 0.195	0.112	0.121	0.197
Making a new chatting acquaintance	0.94 ± 1.09	1 (1–2)	− 0.173	0.105	0.121	0.195
Starting a new paid activity	0.31 ± 1.34	1 (− 1–1)	− 0.216	0.120	0.157	0.213
Belonging to a new group	0.46 ± 1.30	1 (− 1–1)	− 0.215	0.126	0.164	0.192
Interacting with people of different ages and genders	0.79 ± 1.20	1 (1–2)	− 0.197	0.118	0.144	0.194
Starting a new exercise or sport	0.62 ± 1.25	1 (− 1–1)	− 0.172	0.142	0.099	0.171
Starting a new cognitive activity	0.73 ± 1.24	1 (1–2)	− 0.146	0.097	0.188	0.127
Start to participate in social activities	0.67 ± 1.20	1 (− 1–1)	− 0.182	0.133	0.153	0.189
Increasing opportunities to talk to new people	0.70 ± 1.09	1 (1–2)	− 0.185	0.134	0.143	0.201
Starting a new job with no experience	0.19 ± 1.34	1 (− 1–1)	− 0.163	0.096	0.094	0.166

For each item, participants chose their responses from “not at all =  − 2,” “not so much =  − 1,” “somewhat valuable = 1,” and “very valuable = 2.” The values were added to obtain the total score that ranged from − 20 to 20.

PV, perceived value; SD, standardized deviation; GDS, Geriatric Depression Scale-Short Form.

^†^All correlation coefficients have a probability of significance of P < 0.001.

Table 3 shows participants’ characteristics according to the quartiles of the PV scores. Differences were observed in participants’ engagement in physical, cognitive, and social activities (*P* < 0.001); higher PV indicated more engagement in these activities. The ORs and 95% CIs estimated by both crude and adjusted binomial logistic regression analyses for depressive symptoms are shown in Table 4. The PV scores were significantly associated with depressive symptoms. In the adjusted model, compared to the Q1, higher PV scores were independently associated with lower prevalence of depressive symptoms (score range = -20–0, the lowest quartile) (Q2 OR = 0.76, 95% CI = 0.59–0.97; Q3 OR = 0.67, 95% CI = 0.51–0.87; Q4 OR = 0.54, 95% CI = 0.40–0.73; *P* for trend < 0.001).

**Table 3:** Characteristics of participants according to the PV score quartiles.

	Q1 (− 20–0) n = 1315	Q2 (1–8) n = 1354	Q3 (9–13) n = 1386	Q4 (14–20) n = 1211	*P* value
Demographic characteristics					
Age, years	76.1 ± 5.9	74.2 ± 5.4	73.2 ± 5.3	72.8 ± 5.2	< 0.001*
Sex, female (%)	712 (54.1)	707 (52.2)^§^	746 (53.8)	729 (60.2)^‡^	< 0.001^†^
Education, years	11.0 ± 2.3	11.7 ± 2.3	11.9 ± 2.2	12.1 ± 2.2	< 0.001*
Annual household income, ≥ 3 million yen (%)	466 (35.4)^§^	599 (44.2)	707 (51.0)^‡^	634 (52.4)^‡^	< 0.001^†^
Living alone, yes (%)	226 (17.2)^‡^	153 (11.3)^§^	182 (13.1)	167 (13.8)	< 0.001^†^
Alcohol consumption, yes (%)	487 (37.0)^§^	571 (42.2)	625 (45.1)^‡^	507 (41.9)	< 0.001^†^
Smoking, yes (%)	88 (6.7)	91 (6.7)	100 (7.2)	63 (5.2)^§^	0.194^†^
Health factors					
Heart disease, yes (%)	257 (19.5)	248 (18.3)	246 (17.7)	245 (20.2)	0.354^†^
Stroke, yes (%)	97 (7.4)^‡^	79 (5.8)	86 (6.2)	56 (4.6)^§^	0.035^†^
Hypertension, yes (%)	726 (55.2)^‡^	656 (48.4)	641 (46.2)	530 (43.8)^§^	< 0.001^†^
Hyperlipidemia, yes (%)	510 (38.8)	545 (40.3)	540 (39.0)	457 (37.7)	0.631^†^
Diabetes, yes (%)	189 (14.4)	198 (14.6)	187 (13.5)	149 (12.3)	0.325^†^
Pain, yes (%)	610 (46.4)^‡^	566 (41.8)	609 (43.9)	499 (41.2)	0.033^†^
BMI, kg/m^2^	23.4 ± 3.4	23.1 ± 3.0	23.2 ± 3.0	23.2 ± 3.1	0.196*
Cognitive decline, MMSE < 24 (%)	169 (12.9)^‡^	108 (8.0)	72 (5.2)^§^	51 (4.2)^§^	< 0.001^†^
Slow gait, < 1.0 m/s (%)	471 (35.8)^‡^	296 (21.9)	256 (18.5)^§^	183 (15.1)^§^	< 0.001^†^
Engagement in activities					
Physical activity, LA score	14.1 ± 7.9	15.2 ± 7.9	16.1 ± 8.0	17.4 ± 8.7	< 0.001*
Cognitive activity, LA score	9.9 ± 5.8	11.1 ± 6.0	11.7 ± 5.9	12.8 ± 6.4	< 0.001*
Social activity, number	2.3 ± 1.0	2.6 ± 0.9	2.8 ± 0.9	2.9 ± 0.9	< 0.001*
Depressive status					
GDS score, point	3.3 ± 2.7	2.6 ± 2.4	2.2 ± 2.3	1.9 ± 2.1	< 0.001*

BMI, Body Mass Index; MMSE, Mini-Mental State Examination; LA score, Leisure activity score; GDS, Geriatric Depression Scale-Short Form.

*P-values reported from unpaired t-test.

^†^*P*-values obtained by Pearson’s chi square test.

^‡^Statistically significant association by adjusted standardized residual > 1.96 [*P* < 0.05].

^§^Statistically significant association with adjusted standardized residual < 1.96 [*P* < 0.05].

**Table 4:** Crude and adjusted odds ratios and 95% confidence intervals for associations with depressive symptoms.

	Crude model				Adjusted model			
	Odds ratio	95% CI	*P* value	*P* for trend	Odds ratio	95% CI	*P* value	*P* for trend
Perceived value score								
Q1 (− 20–0)	Reference			< 0.001	Reference			< 0.001
Q2 (0–8)	0.54	0.44–0.67	< 0.001		0.76	0.59–0.97	0.026	
Q3 (9–13)	0.42	0.33–0.52	< 0.001		0.67	0.51–0.87	0.003	
Q4 (14–20)	0.30	0.23–0.39	< 0.001		0.54	0.40–0.73	< 0.001	
Demographic characteristics								
Age					1.01	0.99–1.02	0.566	
Sex, female					1.10	0.87–1.39	0.414	
Education					0.97	0.93–1.01	0.187	
Annual household income					0.73	0.60–0.90	0.003	
Living alone					1.55	1.21–1.99	0.001	
Alcohol consumption					0.90	0.72–1.11	0.310	
Smoking					1.78	1.26–2.50	0.001	
Health factors								
Heart disease					1.38	1.10–1.73	0.006	
Stroke					1.82	1.31–2.54	< 0.001	
Hypertension					0.95	0.78–1.17	0.641	
Hyperlipidemia					1.20	0.98–1.47	0.071	
Diabetes					1.13	0.86–1.47	0.377	
Pain					1.65	1.36–2.00	< 0.001	
BMI					0.95	0.92–0.98	0.001	
Cognitive decline					1.60	1.18–2.18	0.003	
Slow gait					1.33	1.07–1.65	0.011	
Engagement in activities								
Physical activity					0.98	0.96–0.99	< 0.001	
Cognitive activity					0.97	0.95–0.98	< 0.001	
Social activity					0.62	0.56–0.69	< 0.001	

CI, confidence interval; BMI, Body Mass Index.

## Discussion

This study aimed to develop a new instrument to measure the PV of adopting new behaviors and clarify whether the PV of adopting new behaviors is associated with depressive symptoms. The internal consistency of the PV questionnaire was acceptable, with a Cronbach’s alpha coefficient of 0.925 and test–retest reliability, as measured by an ICC, of 0.79^43,44^. Higher PV scores were independently associated with a lower risk for depressive symptoms. To the best of our knowledge, this is the first study to clarify the association between the PV of adopting new behaviors and depressive symptoms among older adults.

The prevalence of depressive symptoms in this study was 11.3%. This result was similar to the findings of a report on Japanese older adults and the estimates from a meta-analysis of 24 studies worldwide^5,12^. Differences were found between those with and without depressive symptoms in demographic characteristics, such as age, education, annual household income, living situation, and smoking, health factors, such as history of heart disease and stroke, pain, cognitive decline, and slow gait, and engagement in activities, such as going out for tea or dining with friends and shopping with friends. These characteristics were generally consistent with the results of previous studies in Japan and other countries^9,11,13,35,38^.

Participants with a higher PV of adopting new behaviors were more likely to engage in physical, cognitive, and social activities than those with lower PV. The association between higher PV and higher engagement in activities observed in this study was not unexpected. Values guide, motivate, and influence attitudes and behaviors^45^. Although direct comparisons cannot be made, owing to the lack of reports examining the association between PV and actual engagement in health behaviors, consumer science research has focused on the relationship between PV and attitudes and intentions^22,46,47^. Ma et al. researched the effect of novel and environmentally friendly foods on consumer acceptance and found that PV had a significantly positive effect on attitude, and attitude also had a positive effect on purchase intention^46^. In Li et al.’s study, PV positively affected the behavioral intention of older adults to use remote health management services^47^. These studies showed a positive association between PV, intention, and behavior. In this context, our results can be considered reasonable.

The major finding of this study was that a higher PV is associated with a lower prevalence of depressive symptoms even after adjusting for engagement of activities. The finding of an inverse association between high PV and depressive symptoms was similar to that of previous studies involving adolescents^18,20^. These studies reported a negative association with Schwartz’s concept of openness to change values in all domains of values. Although Schwartz’s concept was not investigated in this study, the PV of adopting new behaviors assessed in this study may have a similar role to that of openness to change values among the various value domains^21^. An independent association between PV and depressive symptoms and actual engagement in the activities can be explained by the hierarchical effect of values, attitudes, and behaviors (ie, the value-attitude-behavior model)^22,48^. In this model, higher value orientation levels lead to higher individual attitude levels, and consequently, higher individual behavioral intentions and actual behavior^48^. For example, the aforementioned study of environmentally friendly foods showed that the higher the consumers’ perception of green value, the higher their attitude towards plant-based meat alternatives; then, the higher the attitude towards plant-based meat alternatives, the higher the purchase behavior of plant-based meat alternatives^46^. These linear correlations may also affect the degree of actual behavior. Thus, the intensity or density of the activity, which cannot be measured by questionnaires asking only about engagement in activities may have been affected by the degree of PV. In addition, those who participated in the activities may have simultaneously participated in activities other than those included in this study, and the high intensity or density of the activity guided by high PV may have suppressed depressive symptoms.

This study had several limitations. First, the study design was cross-sectional; therefore, we could not determine a causal relationship between the PV of adopting new behaviors and depressive symptoms. As depressive symptoms are accompanied by negative emotions and mood, we could not rule out that the PV may be lower because of the presence of depressive symptoms. Future studies should confirm the effects of PV on depressive symptoms and validate the questionnaire. A causal relationship, predictability of future development of depression, and the minimal clinically important difference above indicators should be explored through longitudinal observations or interventions. Second, the participants were assumed to be relatively healthy because they could voluntarily participate in health checkups conducted at community centers. This may have excluded individuals with severe depressive symptoms or other conditions. In addition, our participants were single ethnic and individual regions of people who were relatively mentally healthy, limiting the extent to which our conclusions can be extrapolated to broader populations. Future research with more diverse samples is needed to enhance the external validity and generalizability of our results. Third, engagement in activities was assessed using frequency or dichotomous responses. Future studies should use measurements that can evaluate both the quantity and quality of activities. Fourth, unadjusted variables such as employment and marital status may confound the association between PV and depressive symptoms; however, the E-value for the odds ratio for the Q4 group was 3.18 (CI, 2.12), which indicates the association of unadjusted confounding factor would have to be stronger than 3.18 to overturn the result^49^. Despite these limitations, the major strengths of this study are the large sample size of community-dwelling older adults and comprehensive set of assessments.

## Conclusion

Actual behavior may be influenced by PV, which is inversely associated with depressive symptoms independent of actual engagement in physical, cognitive, and social activities in community-dwelling older adults. Healthcare professionals and those involved in social welfare must pay attention to poor value orientation in older adults, suggesting the need to develop interventions to promote mental health by enhancing PV through education on the benefits of engagement in physical, cognitive, and social activities and sharing success experiences.

## Data Availability

The datasets used and/or analyzed during the present study are available from the corresponding author upon reasonable request.
